# Recurrence patterns stratify post-progression survival after early recurrence of colorectal liver metastases following hepatectomy: a single-center retrospective study

**DOI:** 10.1186/s12957-026-04499-w

**Published:** 2026-07-20

**Authors:** Taiki Mori, Nozomu Sakai, Tsukasa Takayashiki, Shigetsugu Takano, Isamu Hosokawa, Takanori Konishi, Kensuke Suzuki, Hitoe Nishino, Shinichiro Nakada, Masayuki Ohtsuka

**Affiliations:** Department of General Surgery, Graduate School of Medicine, Chiba University, 1-8-1 Inohana, Chuo-ku, Chiba-shi, Chiba, 260-8670 Japan

**Keywords:** Colorectal liver metastases, Early recurrence, Hepatectomy, Post-progression survival, Recurrence pattern

## Abstract

**Background:**

Early recurrence of colorectal liver metastases (CRLM) after hepatectomy is generally considered high-risk; however, the prognosis may differ depending on whether recurrence is confined to a single site or involves multiple sites. This study evaluated post-progression survival (PPS) according to recurrence pattern at first relapse in patients with early recurrence.

**Methods:**

We retrospectively reviewed consecutive patients who underwent initial curative-intent hepatectomy for CRLM at Chiba University Hospital between 2012 and 2022. Patients with recurrence within 12 months were classified as having single-site or multi-site recurrence at first relapse based on the number of involved organs or anatomically distinct compartments. PPS was calculated from the time of first recurrence and analyzed using Kaplan–Meier and Cox regression methods. Post-recurrence treatments, including salvage metastasectomy, were recorded.

**Results:**

Among 151 patients, 78 (51.7%) developed recurrence within 12 months: 43 with single-site and 35 with multi-site recurrence at first relapse. PPS was significantly longer in the single-site group than in the multi-site group (log-rank *p* = 0.016); median PPS was not reached in the single-site group and was 31 months in the multi-site group. On multivariable analysis, multi-site recurrence remained independently associated with poorer PPS (hazard ratio: 1.96, 95% confidence interval: 1.04–3.71; *p* = 0.037). Salvage metastasectomy was performed in 35 patients (44.9%); the proportion was comparable between the single-site and multi-site groups (46.5% vs. 42.9%, respectively; *p* = 0.821).

**Conclusions:**

Among patients with early recurrence after hepatectomy for CRLM, the anatomical extent of the first relapse further stratified PPS. Multi-site recurrence was independently associated with poorer PPS, suggesting that early recurrence is not a single prognostic entity but comprises clinically heterogeneous subgroups.

**Supplementary Information:**

The online version contains supplementary material available at https://doi.org/10.1186/s12957-026-04499-w.

## Background

Colorectal cancer frequently metastasizes to the liver, and hepatectomy offers the best chance for long-term survival in selected patients with colorectal liver metastases (CRLM) [1, 2]. However, recurrence after curative-intent hepatectomy is common and remains the principal driver of long-term mortality. Risk stratification is therefore essential for postoperative counseling, surveillance, and selection of perioperative systemic therapy, and increasingly incorporates molecular features such as *RAS* status [3–7]. Time to recurrence is widely used as a surrogate for tumor aggressiveness [5]. Early recurrence, typically defined as recurrence within 6–12 months, is consistently associated with inferior survival and is often interpreted as evidence of occult systemic disease [8, 9]. 

Nevertheless, early recurrence represents a heterogeneous clinical entity. In clinical practice, some patients present with recurrence confined to a single site, whereas others develop multi-site disease at initial relapse, suggesting differences in disease burden, resectability, and prognosis [10–13]. Despite its clinical relevance, whether the recurrence pattern at first relapse further stratifies outcomes among patients with early recurrence after hepatectomy for CRLM remains unclear [10–13]. 

Recurrence heterogeneity reflects not only timing but also the extent of the first recurrence [12–17]. Patients with single-site recurrence may still be candidates for additional local therapies in select settings, whereas multi-site recurrence more often indicates disseminated disease with limited potential for durable local control [14, 18]. This distinction may be particularly important in early recurrence, which is frequently regarded as a uniformly high-risk group, despite variation in post-recurrence trajectories. Prior studies have often combined recurrence patterns or focused on overall survival, which conflates time to recurrence with outcomes after recurrence [8, 11, 19]. Thus, a recurrence-centered endpoint may better capture the clinical heterogeneity of early recurrence [20]. 

Interpretation of salvage therapies is also complicated by treatment selection and immortal time biases, as only patients who survive and remain resectable are eligible [21, 22]. Therefore, rather than focusing on treatment, we examined the anatomical extent of the disease at first recurrence. We hypothesized that recurrence pattern—single-site versus multi-site—could provide a simple and clinically meaningful prognostic stratification. Accordingly, we evaluated its association with post-progression survival (PPS) among patients with early recurrence after curative-intent hepatectomy for CRLM.

## Methods

### Study design and patient population

This retrospective cohort study included consecutive patients who underwent initial curative-intent hepatectomy for CRLM at Chiba University Hospital between January 2012 and December 2022. Curative intent was defined as complete macroscopic treatment of all liver metastases, with no unresected extrahepatic disease. Patients were excluded if hepatectomy was performed for non-colorectal primary tumors, if macroscopic residual disease (R2) remained, or if postoperative follow-up data were unavailable. Patients undergoing combined local treatments (e.g., hepatectomy with thermal ablation) were included if all lesions were treated with curative intent.

The analysis focused on patients who developed recurrence within 12 months. Postoperative surveillance generally included physical examination and tumor markers every 3–6 months and contrast-enhanced computed tomography of the chest, abdomen, and pelvis at similar intervals during the first 2 years, followed by longer intervals thereafter, which is broadly consistent with surveillance strategies reported in prior studies and systematic reviews [23]. Surveillance was not strictly protocolized and varied by clinical judgment. Follow-up was updated to December 31, 2025.

The study protocol was approved by the Institutional Review Board of Chiba University Hospital (approval code: HK202507-08). The requirement for individual informed consent was waived due to the retrospective design and the use of de-identified data. This study was reported in accordance with the STROBE statement for observational studies.

### Exposure assessment

The primary exposure was the recurrence pattern at first relapse within 12 months, classified as single-site or multi-site. Recurrence was identified by imaging and/or pathological confirmation when available; otherwise, it was determined based on serial imaging and multidisciplinary assessment.

Recurrence patterns were classified according to the anatomical extent of disease at first relapse. Each involved organ or anatomically distinct extra-organ compartment was considered a single anatomical site. Multiple lesions confined to the same organ were classified as single-site recurrence, irrespective of their number or distribution within that organ. Thus, multifocal intrahepatic recurrence involving different liver segments was classified as single-site recurrence. Recurrence involving two or more organs or anatomically distinct compartments was classified as multi-site recurrence. Accordingly, intrahepatic recurrence accompanied by hepatic hilar lymph node metastasis was classified as multi-site recurrence because the liver and regional lymph nodes were considered anatomically distinct sites. Single-site recurrences were further categorized as liver, lung, or other for descriptive purposes.

### Outcome assessment

The primary endpoint was PPS, defined as the interval from the first recurrence to death from any cause or to the last follow-up. Surviving patients were censored at the last follow-up.

Post-recurrence treatments, including salvage metastasectomy, were recorded. Salvage metastasectomy was defined as repeat curative-intent resection of recurrent disease, including repeat hepatectomy, pulmonary metastasectomy, or resections at multiple sites as part of the same treatment strategy [18]. 

### Other variables

Baseline clinicopathological variables were extracted from medical records and operative and pathological reports, including age, sex, primary tumor location, primary lymph node status, synchronous versus metachronous liver metastasis, number of liver metastases, largest tumor size, bilobar involvement, major hepatectomy (≥ 3 segments), and resection margin status. Molecular tumor data, including *RAS* and *BRAF* mutation status, were recorded when available.

### Statistical analysis

Continuous variables are presented as medians (interquartile ranges), and categorical variables are presented as counts (percentages). Baseline characteristics were compared between the single-site and multi-site recurrence groups using the Mann–Whitney U test for continuous variables and the chi-square test or Fisher’s exact test for categorical variables, as appropriate.

PPS was estimated using the Kaplan–Meier method and compared with the log-rank test. Cox proportional hazards models were used to estimate hazard ratios (HRs) and 95% confidence intervals (CIs). Covariates for multivariable analysis were selected a priori based on clinical relevance and the number of events. A two-sided *p* < 0.05 was considered statistically significant. Analyses were performed using JMP (SAS Institute, Cary, NC, USA) and R (R Foundation for Statistical Computing, Vienna, Austria).

## Results

### Patient cohort and early recurrence patterns

**Fig. 1:**
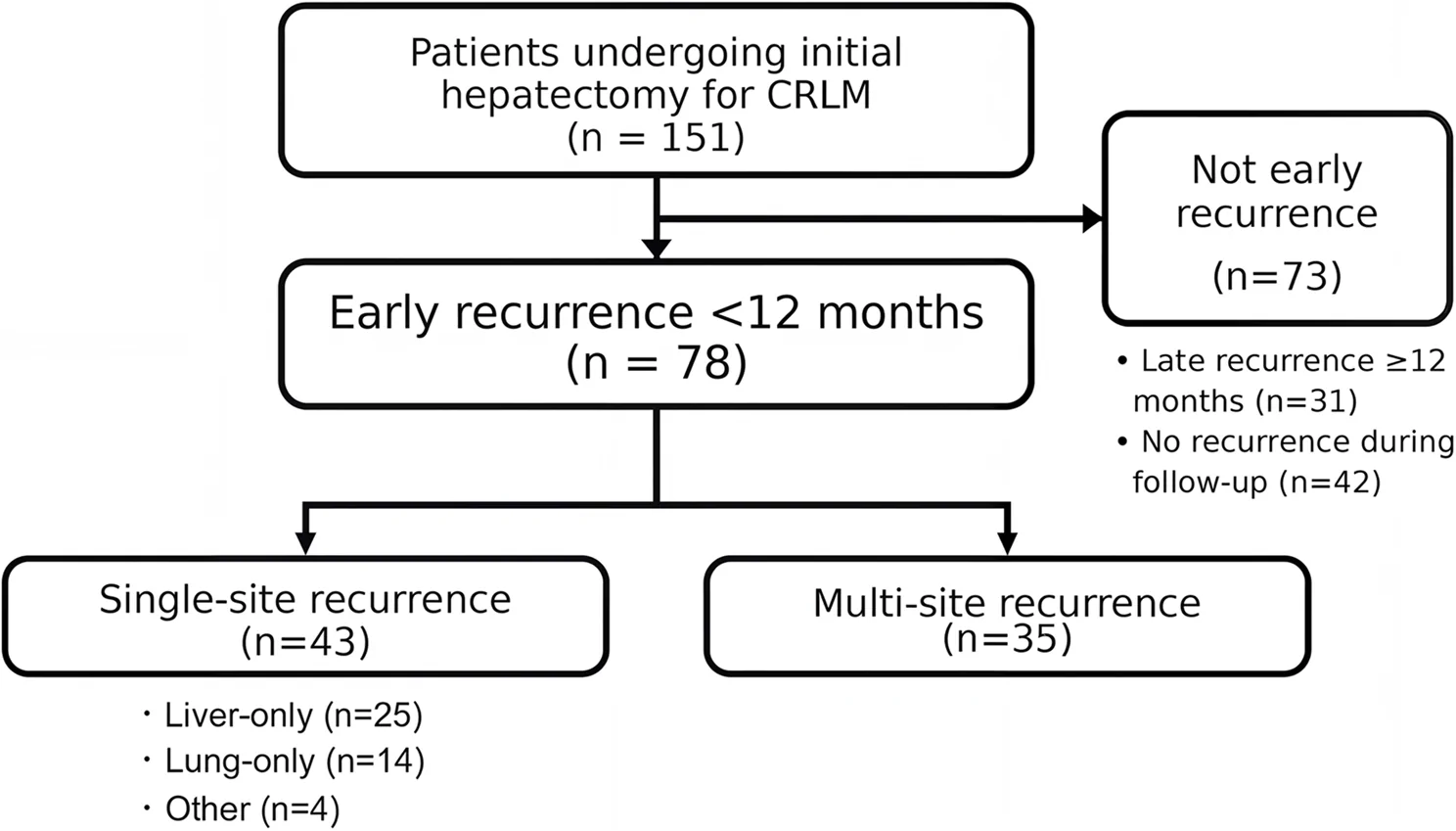
Study flow diagram and stratification of early recurrence into single-site versus multi-site recurrence. Patients who underwent initial hepatectomy for Colorectal Liver Metastasis (CRLM) were screened. Those who developed early recurrence within 12 months after hepatectomy were identified and further stratified by the extent of recurrence at the first relapse: single-site or multi-site. The diagram summarizes the cohort selection and classification of patients with early recurrence into the single-site and multi-site groups. The organ-specific first-recurrence patterns are shown in Supplementary Figure S2

A total of 151 patients underwent initial hepatectomy for CRLM. Early recurrence within 12 months occurred in 78 patients (51.7%), while 73 patients were classified as not having early recurrence, including 31 with late recurrence (≥ 12 months) and 42 without recurrence during follow-up (Fig. 1).

For the primary analysis, patients with early recurrence were stratified according to the anatomical extent of disease at first relapse into single-site and multi-site recurrence groups. Among the 78 patients with early recurrence, 43 (55.1%) had single-site recurrence and 35 (44.9%) had multi-site recurrence. In the single-site recurrence group, the first recurrence site was the liver in 25 patients (58.1%), the lungs in 14 (32.6%), and other sites in 4 (9.3%). Salvage metastasectomy was performed in 35 patients (44.9%) overall, including 20 of 43 patients (46.5%) with single-site recurrence and 15 of 35 patients (42.9%) with multi-site recurrence. The proportion of patients undergoing salvage metastasectomy did not differ significantly between the two groups (*p* = 0.821, Fisher’s exact test).

### Baseline characteristics according to recurrence pattern in early recurrence

Table 1 summarizes the baseline clinicopathological characteristics, stratified by first recurrence pattern, among patients with early recurrence. Overall, the single-site and multi-site groups were generally comparable, with no significant differences in age, sex, primary tumor location, primary lymph node status, synchronous liver metastasis, number of liver metastases, bilobar disease, resection margin status, and *RAS/BRAF* mutation status.

**Table 1:** Baseline characteristics and salvage metastasectomy according to first recurrence pattern

Variable	Single-site recurrence (*n* = 43)	Multi-site recurrence (*n* = 35)	*p* value
Baseline characteristics			
Age, years	69 [61–76]	69 [59–78]	0.960
Male sex, *n* (%)	28 (65.1)	22 (62.9)	0.812
Primary tumor location, *n* (%)	Right-sided 10 (23.3) / Left-sided 18 (41.9) / Rectal 14 (32.6)	Right-sided 6 (17.1) / Left-sided 9 (25.7) / Rectal 19 (54.3)	0.264
Primary lymph node status, *n* (%)	N0 10 (23.3) / *N* + 32 (74.4)	N0 9 (25.7) / *N* + 25 (71.4)	0.797
Synchronous liver metastasis, *n* (%)	20 (47.6)	18 (51.4)	0.821
Number of liver metastases, *n*	2 [1–4]	3 [1–4]	0.812
Largest tumor size, cm	2.5 [1.7–3.5]	3.2 [2.2–4.2]	0.175
Bilobar disease, *n* (%)	18 (41.9)	18 (51.4)	0.498
Major hepatectomy (≥ 3 segments), *n* (%)	6 (14.0)	11 (31.4)	0.095
R1 resection margin, *n* (%)	7 (16.3)	10 (28.6)	0.271
RAS mutant*, *n* (%)	17 (44.7)	14 (48.3)	0.809
BRAF mutant*, *n* (%)	2 (6.1)	1 (5.9)	1.000
Post-recurrence treatment			
Salvage metastasectomy, *n* (%)	20 (46.5)	15 (42.9)	0.821

Data are presented as medians [interquartile range] or *n* (%). *P* values were calculated using the Mann–Whitney U test for continuous variables and Fisher’s exact test or the chi-square test, as appropriate, for categorical variables. Major hepatectomy was defined as resection of ≥ 3 Couinaud segments. R1 indicates a microscopically positive resection margin. Patients who underwent R2 resection were excluded from the comparison of resection margin status. Primary tumor location was unavailable in two patients (single-site, *n* = 1; multi-site, *n* = 1), primary lymph node status in two patients (single-site, *n* = 1; multi-site, *n* = 1), and timing of liver metastasis in one patient in the single-site recurrence group; these were handled as “unknown” categories when calculating p values. *RAS and *BRAF* mutation rates are shown for patients with available molecular test results in each group (*RAS*, *n* = 67; *BRAF*, *n* = 50). Salvage metastasectomy included repeat hepatectomy, pulmonary metastasectomy, and resection of other recurrent metastatic lesions, as appropriate

The multi-site group showed a trend toward larger maximum tumor size (3.2 [2.2–4.2] vs. 2.5 [1.7–3.5] cm) and higher rates of major hepatectomy (31.4% vs. 14.0%), although these differences were not statistically significant.

### PPS according to the recurrence pattern

**Fig. 2:**
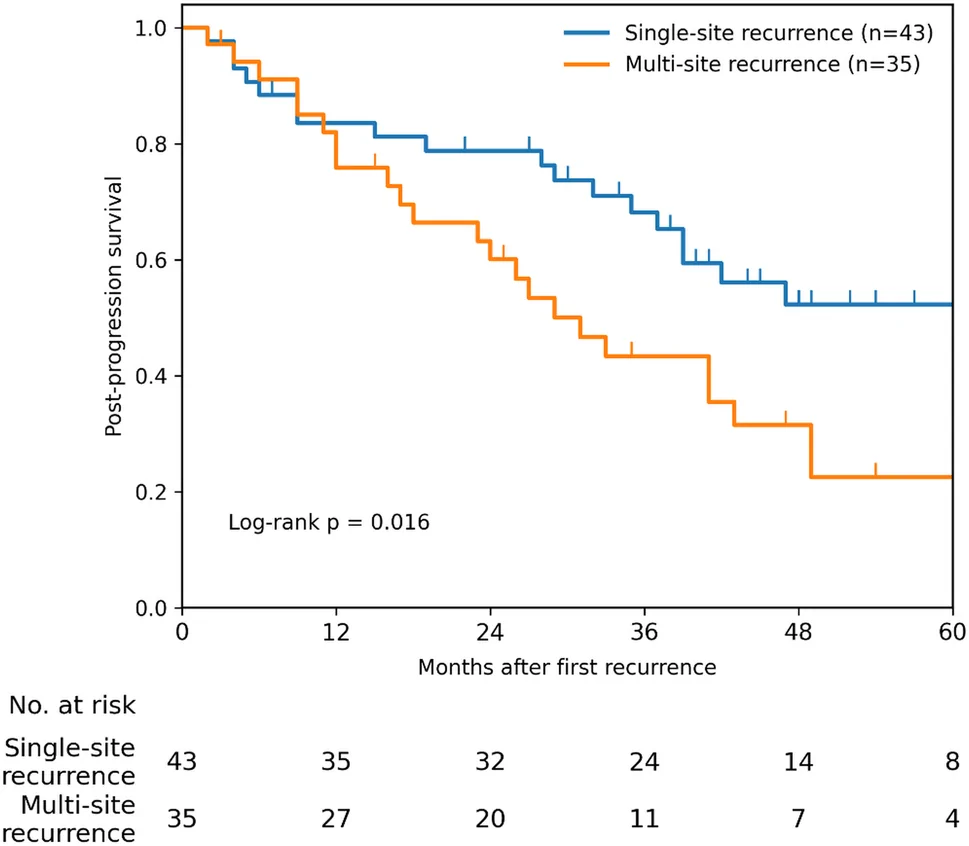
Kaplan–Meier curves for Post-Progression Survival (PPS) according to single-site versus multi-site recurrence among patients with early recurrence. Kaplan–Meier curves of PPS are shown for patients who developed early recurrence within 12 months after initial hepatectomy for Colorectal Liver Metastases (CRLM), stratified as having single-site or multi-site recurrence at first relapse. Patients with single-site recurrence had a significantly longer PPS than those with multi-site recurrence (log-rank *p* = 0.016). The median PPS was not reached in the single-site recurrence group, whereas it was 31 months in the multi-site recurrence group. Numbers at risk are shown below the plot

PPS differed significantly by recurrence pattern at first relapse (Fig. 2). Patients with single-site recurrence had significantly longer PPS than those with multi-site recurrence (log-rank *p* = 0.016). Median PPS was not reached in the single-site recurrence group, but it was 31 months in the multi-site recurrence group. Survival curves separated early and remained distinct throughout follow-up.

### Cox regression analysis for PPS in early recurrence

On univariable analysis (Table 2), multi-site recurrence (hazard ratio [HR]: 2.08, 95% confidence interval [CI]: 1.13–3.85; *p* = 0.019), number of liver metastases (HR: 1.09, 95% CI: 1.02–1.18; *p* = 0.018), and R1 resection margin (HR: 2.07, 95% CI: 1.05–4.07; *p* = 0.036) were associated with worse PPS.

**Table 2:** Cox regression analyses for post-progression survival among patients with early recurrence (< 12 months)

Variable	Univariable HR (95% CI)	*p* value	Multivariable HR (95% CI)	*p* value
Multi-site recurrence (vs. single-site)	2.08 (1.13–3.85)	0.019	1.96 (1.04–3.71)	0.037
Primary lymph node status, N+ (vs. N0)	1.49 (0.69–3.24)	0.310	1.26 (0.57–2.80)	0.567
Number of liver metastases	1.09 (1.02–1.18)	0.018	1.07 (0.99–1.16)	0.096
R1 resection margin (vs. R0)	2.07 (1.05–4.07)	0.036	1.59 (0.76–3.32)	0.221

Hazard Ratios (HRs) with 95% Confidence Intervals (CIs) are presented. Post-progression Survival (PPS) was defined as the interval from the date of the first recurrence after the initial hepatectomy to death from any cause or the last follow-up. The multivariable Cox model included recurrence pattern, primary lymph node status, number of liver metastases, and resection margin status, and was fitted using complete cases with available covariates. Patients with missing data on primary lymph node status were excluded from the multivariable analysis. The resection margin status was analyzed as R1 versus R0; patients who underwent R2 resection were excluded

In the multivariable model including recurrence pattern, primary lymph node status, number of liver metastases, and resection margin status, multi-site recurrence remained independently associated with poorer PPS (HR: 1.96, 95% CI: 1.04–3.71; *p* = 0.037). The number of liver metastases showed a borderline association with PPS (HR: 1.07, 95% CI: 0.99–1.16; *p* = 0.096), whereas primary lymph node status and R1 resection margin were not significant.

### Supplementary analyses

Overall survival after initial hepatectomy was lower in patients with early recurrence (< 12 months) than in those with late recurrence (≥ 12 months) (Figure S1).

An exploratory organ-specific analysis of first recurrence among patients with early recurrence is shown in Figure S2; lung-only recurrence appeared to have the most favorable PPS, although the difference was not statistically significant. A 3-month landmark analysis evaluating salvage metastasectomy (Figure S3) showed a similar trend toward improved PPS, but it was not statistically significant. *RAS* mutation status was available for 119 patients (78.8%), and mutations were present in 44 (37.0%). Among recurrent cases with available data, *RAS* mutations were more frequent in early than in late recurrence (31/67 [46.3%] vs. 6/24 [25.0%]; *p* = 0.091) (Table S3 and Figure S4).

## Discussion

In this single-institution cohort of patients with early recurrence within 12 months after hepatectomy for CRLM, prognosis after recurrence differed according to whether the first relapse was confined to a single site or involved multiple sites. Importantly, the main finding was not merely that multi-site recurrence was associated with poorer survival, but that early recurrence itself comprised prognostically distinct subgroups according to the anatomical extent of the first relapse. Patients with single-site recurrence had a significantly longer PPS than those with multi-site recurrence, and this association remained significant after adjustment for primary lymph node status, number of liver metastases, and resection margin status. The comparable rates of salvage metastasectomy between the two groups indicate that the observed difference in PPS was unlikely to be explained solely by the overall frequency of salvage surgery. These findings suggest that early recurrence is not a uniform clinical entity; rather, the anatomical extent at first relapse captures clinically relevant heterogeneity in post-recurrence outcomes, consistent with prior reports linking recurrence pattern to survival after CRLM resection [10, 11, 15, 24]. 

Early recurrence is often considered a surrogate of aggressive tumor biology and occult systemic disease [25]. However, our results suggest that this view is incomplete. While time to recurrence remains useful for postoperative counseling and surveillance, prognosis after recurrence is also strongly influenced by disease extent at relapse. By focusing on PPS, this study more directly reflects post-recurrence decision-making than overall survival. As expected, early recurrence was also associated with inferior overall survival compared with late recurrence (Figure S1). However, overall survival reflects both time to recurrence and post-recurrence course, supporting PPS as a more adequate endpoint in this context [19, 26]. 

Several mechanisms may explain the longer PPS observed in single-site early recurrence. Disease confined to one anatomical site may be more amenable to durable local control, including repeat hepatectomy or pulmonary metastasectomy in selected patients^,^ [14, 18, 27, 28] whereas multi-site recurrence more often reflects a greater systemic tumor burden and limited potential for durable local control [22]. Differences in detectability, resectability, and metastatic organotropism may also contribute.

Salvage metastasectomy was performed in 44.9% of patients with early recurrence. Contrary to the possibility that the survival difference was primarily attributable to greater use of salvage surgery in patients with anatomically limited recurrence, the proportion undergoing salvage metastasectomy was similar between the single-site and multi-site groups. This finding suggests that the observed difference in PPS was unlikely to be explained solely by differences in the overall frequency of salvage surgery and supports the prognostic relevance of the anatomical extent of recurrence. Nevertheless, the present analysis did not account for the site, extent, timing, or completeness of salvage resection, and treatment selection was inherently influenced by disease distribution, technical resectability, patient condition, and multidisciplinary judgment. The supplementary landmark analysis stratified by salvage metastasectomy status provides additional exploratory information but should be interpreted cautiously because of treatment-selection and time-related biases [21, 22]. 

Our findings may have practical implications for post-recurrence stratification of patients with early relapse after hepatectomy for CRLM. Although patients with early recurrence are often grouped as having a poor prognosis, our results suggest that this classification alone may be overly simplistic. Patients with single-site recurrence, particularly those with disease confined to the liver or lungs, may still have an opportunity for prolonged survival. However, single-site recurrence should not be interpreted as uniformly favorable disease, as some patients still have multiple lesions or otherwise unresectable recurrences within a single organ. This classification may serve as a practical first step for post-recurrence stratification, helping to identify patients who need careful multidisciplinary assessment for possible salvage local treatment. In contrast, multi-site recurrence appears to indicate a less favorable post-recurrence course and may warrant prioritizing systemic therapy, with local intervention reserved for highly selected situations [14, 18, 27, 29]. 

Although our molecular analyses were exploratory, the observed trend is broadly consistent with prior reports that suggest associations among *RAS* mutation status, recurrence pattern, and salvageability, with variable prognostic impact depending on disease timing [16, 17, 30]. 

This study has several limitations. First, this was a retrospective, single-institution study and was therefore subject to residual confounding. Second, the sample size was modest, particularly for the subgroup and molecular analyses. Third, post-recurrence treatment was not standardized and may have varied according to patient condition, disease distribution, technical resectability, physician judgment, and changes in systemic therapy over time. Although the overall proportion of patients undergoing salvage metastasectomy did not differ significantly between the recurrence-pattern groups, residual treatment-related confounding cannot be excluded. The type, extent, timing, and completeness of salvage resection, as well as subsequent systemic treatment, may have differed between patients and were not fully incorporated into the multivariable model. Moreover, although salvage metastasectomy was associated with longer PPS, treatment-selection and time-related biases could not be fully eliminated, and this association should not be interpreted as a direct treatment effect. Similarly, the absence of a statistically significant difference in salvage metastasectomy rates should not be interpreted as evidence of equivalence between the recurrence-pattern groups, given the limited sample size. Finally, molecular testing, including RAS and BRAF mutation status, was not available for all patients; therefore, these analyses should be considered exploratory.

Despite these limitations, this study had several strengths. It was based on a consecutive surgical cohort, used a clinically intuitive classification of early recurrence as single-site versus multi-site disease, and focused on the PPS to more directly characterize the post-recurrence course. These findings support the concept that patients with early recurrence after hepatectomy for CRLM should not automatically be regarded as a homogeneous group with a poor prognosis. Larger multi-institutional studies are needed to validate this recurrence pattern-based stratification and to determine how the anatomical extent at first recurrence can be integrated with molecular and radiologic markers to improve the prediction of salvageability and long-term outcomes.

## Conclusion

Among patients with early recurrence after hepatectomy for CRLM, the recurrence pattern at first relapse further stratified PPS. Single-site recurrence was associated with significantly better PPS than multi-site recurrence, suggesting that early recurrence is clinically heterogeneous rather than uniformly aggressive. Classifying the first recurrence as single-site or multi-site may help identify patients with different post-recurrence prognoses, supporting multidisciplinary treatment planning.

## Supplementary Information

Additional file 1: Supplementary Tables and Figures. This file contains Supplementary Tables S1–S3 and Supplementary Figures S1–S4.

## Data Availability

The datasets generated and/or analyzed during the current study are available from the corresponding author on reasonable request.

## Abbreviations

CRLM: Colorectal liver metastases
PPS: Post-progression survival
HR: Hazard ratio
CI: Confidence interval
IRB: Institutional Review Board

## References

[1] Choti MA, Sitzmann JV, Tiburi MF, et al. Trends in long-term survival following liver resection for hepatic colorectal metastases. Ann Surg. 2002;235:759–66. 10.1097/00000658-200206000-00002 PMC1422504 12035031

[2] Tomlinson JS, Jarnagin WR, DeMatteo RP, et al. Actual 10-year survival after resection of colorectal liver metastases defines cure. J Clin Oncol. 2007;25:4575–80. 10.1200/JCO.2007.11.0833 17925551

[3] Fong Y, Fortner J, Sun RL, Brennan MF, Blumgart LH. Clinical score for predicting recurrence after hepatic resection for metastatic colorectal cancer: analysis of 1001 consecutive cases. Ann Surg. 1999;230:309 – 18;discussion 318. 10.1097/00000658-199909000-00004 PMC1420876 10493478

[4] Nordlinger B, Guiguet M, Vaillant JC, Balladur P, Boudjema K, Bachellier P. Surgical resection of colorectal carcinoma metastases to the liver: a prognostic scoring system to improve case selection, based on 1568 patients. Cancer. 1996;77:1254–62. 8608500

[5] Rees M, Tekkis PP, Welsh FKS, O’Rourke T, John TG. Evaluation of long-term survival after hepatic resection for metastatic colorectal cancer: a multifactorial model of 929 patients. Ann Surg. 2008;247:125–35. 10.1097/SLA.0b013e31815aa2c2 18156932

[6] Shindoh J. Risk stratification and goal of systemic therapy for successful surgical management of colorectal liver metastases: oncological optimization. J Hepatobiliary Pancreat Sci. 2021;28:461–9. 10.1002/jhbp.964 33797853

[7] Takematsu T, Mima K, Hayashi H, et al. RAS mutation status in combination with the JSHBPS nomogram may be useful for preoperative identification of colorectal liver metastases with high risk of recurrence and mortality after hepatectomy. J Hepatobiliary Pancreat Sci. 2024;31:69–79. 10.1002/jhbp.1389 37897144

[8] Tian Y, Wang Y, Wen N, Wang S, Li B, Liu G. Prognostic factors associated with early recurrence following liver resection for colorectal liver metastases: a systematic review and meta-analysis. BMC Cancer. 2024;24:426. 10.1186/s12885-024-12162-4 PMC11000331 38584263

[9] Imai K, Allard MA, Benitez CC, et al. Early recurrence after hepatectomy for colorectal liver metastases: what optimal definition and what predictive factors? Oncologist. 2016;21:887–94. 10.1634/theoncologist.2015-0468 PMC4943389 27125753

[10] de Jong MC, Pulitanò C, Ribero D, et al. Rates and patterns of recurrence following curative intent surgery for colorectal liver metastasis: an international multi-institutional analysis of 1669 patients. Ann Surg. 2009;250:440–8. 10.1097/SLA.0b013e3181b4539b 19730175

[11] Wong GYM, Mol B, Bhimani N, et al. Recurrence patterns predict survival after resection of colorectal liver metastases. ANZ J Surg. 2022;92:2149–56. 10.1111/ans.17835 PMC9544678 35666139

[12] Sawada Y, Sahara K, Endo I, et al. Long-term outcome of liver resection for colorectal metastases in the presence of extrahepatic disease: a multi-institutional Japanese study. J Hepatobiliary Pancreat Sci. 2020;27:810–8. 10.1002/jhbp.810 32713080

[13] Bilobar CRC, Liver Metastases Research Collaborative. Recurrence patterns and post-recurrence survival of patients with bilobar colorectal liver metastases: international multicentre retrospective study. Br J Surg. 2025;112:znaf003. 10.1093/bjs/znaf003 40037797

[14] Boyev A, Tzeng CD, Maki H, et al. Local therapy improves survival for early recurrence after resection of colorectal liver metastases. Ann Surg Oncol. 2024;31:2547–56. 10.1245/s10434-023-14806-4 38148351

[15] D’Angelica M, Kornprat P, Gonen M, et al. Effect on outcome of recurrence patterns after hepatectomy for colorectal metastases. Ann Surg Oncol. 2011;18:1096–103. 10.1245/s10434-010-1409-1 21042942

[16] Okuno M, Goumard C, Kopetz S, et al. RAS mutation is associated with unsalvageable recurrence following hepatectomy for colorectal cancer liver metastases. Ann Surg Oncol. 2018;25:2457–66. 10.1245/s10434-018-6517-3 29786130

[17] Vauthey JN, Zimmitti G, Kopetz SE, et al. RAS mutation status predicts survival and patterns of recurrence in patients undergoing hepatectomy for colorectal liver metastases. Ann Surg. 2013;258:619–26. discussion 626. 10.1097/SLA.0b013e3182a5025a PMC3856211 24018645

[18] Oba M, Hasegawa K, Shindoh J, et al. Survival benefit of repeat resection of successive recurrences after the initial hepatic resection for colorectal liver metastases. Surgery. 2016;159:632–40. 10.1016/j.surg.2015.09.003 26477476

[19] Ecker BL, Lee J, Saadat LV, et al. Recurrence-free survival versus overall survival as a primary endpoint for studies of resected colorectal liver metastasis: a retrospective study and meta-analysis. Lancet Oncol. 2022;23:1332–42. 10.1016/S1470-2045(22)00506-X 36058227

[20] Angelsen JH, Horn A, Eide GE, et al. Predictive factors for time to recurrence, treatment and post-recurrence survival after liver resection for colorectal liver metastases. World J Surg Oncol. 2015;13:328. 10.1186/s12957-015-0738-8 PMC4668655 26631156

[21] Agarwal P, Moshier E, Ru M, et al. Immortal time bias in observational studies of time-to-event outcomes: assessing effects of postmastectomy radiation therapy using the national cancer database. Cancer Control. 2018;25:1073274818789355. 10.1177/1073274818789355 PMC6053873 30021466

[22] Jones M, Fowler R. Immortal time bias in observational studies of time-to-event outcomes. J Crit Care. 2016;36:195–9. 10.1016/j.jcrc.2016.07.017 27546771

[23] Jones A, Findlay A, Knight SR, et al. Follow up after surgery for colorectal liver metastases: a systematic review. Eur J Surg Oncol. 2023;49:107103. 10.1016/j.ejso.2023.107103 37890234

[24] Sakai N, Furukawa K, Takayashiki T, Kuboki S, Takano S, Ohtsuka M. Recurrence patterns and their effects on clinical outcomes after R1 resection of colorectal liver metastases: a propensity score-matched analysis. Langenbecks Arch Surg. 2021;406:2739–47. 10.1007/s00423-021-02096-x 34031728

[25] Viganò L, Gentile D, Galvanin J, et al. Very early recurrence after liver resection for colorectal metastases: incidence, risk factors, and prognostic impact. J Gastrointest Surg. 2022;26:570–82. 10.1007/s11605-021-05123-w 34508293

[26] Oba M, Hasegawa K, Matsuyama Y, et al. Discrepancy between recurrence-free survival and overall survival in patients with resectable colorectal liver metastases: a potential surrogate endpoint for time to surgical failure. Ann Surg Oncol. 2014;21:1817–24. 10.1245/s10434-014-3504-1 24499828

[27] Butte JM, Gönen M, Allen PJ, et al. Recurrence after partial hepatectomy for metastatic colorectal cancer: potentially curative role of salvage repeat resection. Ann Surg Oncol. 2015;22:2761–71. 10.1245/s10434-015-4370-1 PMC4780326 25572686

[28] Folkerts AD, Janczewski LM, Merkow RP. Liver-directed therapies for colorectal liver metastases. Cancer. 2025;131:e70097. 10.1002/cncr.70097 PMC12548511 41070808

[29] Kobayashi K, Inoue Y, Oba A, et al. Strategies for recurrent colorectal liver metastases based on prognostic factors and resectability: potential benefit of multidisciplinary treatment. Ann Surg Oncol. 2025;32:1729–41. 10.1245/s10434-024-16491-3 39580378

[30] Sakai N, Furukawa K, Takayashiki T, Kuboki S, Takano S, Ohtsuka M. Differential effects of KRAS mutational status on long-term survival according to the timing of colorectal liver metastases. BMC Cancer. 2021;21:412. 10.1186/s12885-021-08144-5 PMC8048293 33858364

