# Supplementary material for: Recurrence patterns stratify post-progression survival after early recurrence of colorectal liver metastases following hepatectomy: a single-center retrospective study
Source: World J Surg Oncol. 2026 Jul 20;24:402. doi: 10.1186/s12957-026-04499-w (PMC13673725; doi:10.1186/s12957-026-04499-w)
Supplement: Supplementary file 1 [file 12957_2026_4499_MOESM1_ESM.docx]

Additional file

Title:
Recurrence Patterns Stratify Post-Progression Survival After Early Recurrence of Colorectal Liver Metastases Following Hepatectomy: A Single-Center Retrospective Study

Authors:
Taiki Mori, MD; Masayuki Ohtsuka, MD, PhD; et al.

Affiliation:
Department of General Surgery, Graduate School of Medicine, Chiba University, Chiba, Japan

This file contains supplementary tables and figures.

## Supplementary Table S1

Baseline characteristics of the entire cohort (n = 151)

| **Variable** | **All patients (n = 151)** |
| --- | --- |
| Age, years | 69.0 [60.0–74.8] |
| Male sex, n (%) | 98 (64.9) |
| Primary tumor location, n (%) | Right-sided 26 (17.2) / Left-sided 64 (42.4) / Rectal 55 (36.4) |
| Primary lymph node status, n (%) | N0 48 (31.8) / N+ 98 (64.9) |
| Synchronous liver metastasis, n (%) | 74 (49.0) |
| Number of liver metastases, n | 2 [1–4] |
| Largest tumor size, cm | 2.6 [1.7–4.0] |
| Bilobar disease, n (%) | 59 (39.1) |
| Major hepatectomy (≥3 segments), n (%) | 24 (15.9) |
| R1 resection margin, n (%) | 28 (18.5) |
| *RAS* mutant* (n = 119) | 44 (37.0) |
| *BRAF* mutant* (n = 93) | 4 (4.3) |

Legend
Supplementary Table S1. Baseline characteristics of the entire cohort undergoing initial hepatectomy for colorectal liver metastases (CRLM) (n = 151).

Footnote

Data are presented as median [interquartile range] or n (%). Primary lymph node status was unavailable in 5 patients. *RAS* and *BRAF* mutation rates are shown among patients with available molecular testing (*RAS*, n = 119; *BRAF*, n = 93).

## Supplementary Table S2

Comparison between early and late recurrence

| **Variable** | **Early recurrence**  **(<12 months) (n = 78)** | **Late recurrence**  **(≥12 months) (n = 31)** | **p value** |
| --- | --- | --- | --- |
| Age, years | 69.0 [61.0–76.8] | 70.0 [62.5–74.0] | 0.989 |
| Male sex, n (%) | 50 (64.1) | 24 (77.4) | 0.256 |
| Primary tumor location, n (%) | Right-sided 15 (19.2) / Left-sided 28 (35.9) / Rectal 32 (41.0) | Right-sided 4 (12.9) / Left-sided 12 (38.7) / Rectal 13 (41.9) | 0.828 |
| Primary lymph node status, n (%) | N0 19 (25.0) / N+ 57 (75.0) | N0 14 (48.3) / N+ 15 (51.7) | **0.033** |
| Synchronous liver metastasis, n (%) | 38 (48.7) | 18 (58.1) | 0.404 |
| Number of liver metastases, n (%) | 3 [1–4] | 2 [1–4] | 0.544 |
| Largest tumor size, cm | 3.0 [1.8–4.1] | 2.2 [1.6–3.7] | 0.286 |
| Bilobar disease, n (%) | 36 (46.2) | 10 (32.3) | 0.204 |
| Major hepatectomy (≥3 segments), n (%) | 17 (21.8) | 3 (9.7) | 0.177 |
| R1 resection margin, n (%) | 19 (24.4) | 4 (12.9) | 0.297 |
| *RAS* mutant*, n (%) | 31 (46.3) | 6 (25.0) | 0.091 |
| *BRAF* mutant*, n (%) | 3 (6.0) | 1 (5.3) | 1.000 |

Legend
Supplementary Table S2. Comparison of clinicopathological characteristics between patients with early recurrence (<12 months) and late recurrence (≥12 months) after hepatectomy for colorectal liver metastases among those who developed recurrence (n = 109).

Footnote
Data are presented as median [interquartile range] or n (%). P values were calculated using the Mann–Whitney U test for continuous variables and the χ² test or Fisher’s exact test for categorical variables, as appropriate. Primary tumor location was unavailable in 5 patients (early, n = 3; late, n = 2) and was handled as an “unknown” category for p value calculation. Primary lymph node status was unavailable in 4 patients (early, n = 2; late, n = 2). **RAS* and *BRAF* mutation rates are shown among patients with available molecular testing in each group (*RAS*: early, n = 67; late, n = 24; *BRAF*: early, n = 50; late, n = 19).

## Supplementary Table S3

Association between *RAS* mutation status and recurrence timing and site-specific recurrence patterns (tested patients)

a. *RAS* status by recurrence timing

|  | ***RAS* mutant** | ***RAS* wild-type** | **Total** | **p value**  **(Fisher)** |
| --- | --- | --- | --- | --- |
| Early recurrence (<12 months) | 31  (46.3) | 36  (53.7) | 67 | 0.091 |
| Late recurrence (≥12 months) | 6  (25.0) | 18  (75.0) | 24 |  |

b. *RAS* status by first recurrence site among early recurrences

|  | ***RAS* mutant** | ***RAS* wild-type** | **Total** | **p value**  **(Fisher)** |
| --- | --- | --- | --- | --- |
| Liver-only (early) | 10  (43.5) | 13  (56.5) | 23 | 0.313 |
| Lung-only (early) | 9  (64.3) | 5  (35.7) | 14 |  |

c. *RAS* status by first recurrence site among all recurrences

|  | ***RAS* mutant** | ***RAS* wild-type** | **Total** | **p value**  **(Fisher)** |
| --- | --- | --- | --- | --- |
| Liver-only (all) | 11  (35.5) | 20  (64.5) | 31 | 0.255 |
| Lung-only (all) | 10  (52.6) | 9  (47.4) | 19 |  |

Legend

Supplementary Table S3. *RAS* status by recurrence timing and first recurrence site among tested patients.

a. *RAS* status by recurrence timing

b. *RAS* status by first recurrence site among early recurrences

c. *RAS* status by first recurrence site among all recurrences

Footnote
Data are presented as n (%). P values were calculated using Fisher’s exact test. Denominators indicate the number of patients with available *RAS* status in each subgroup. Among patients with early recurrence, organ-specific analyses were restricted to the 67 patients with available *RAS* data.

## Supplementary Figure S1

Overall survival according to early and late recurrence after hepatectomy

Legend
Kaplan–Meier curves of overall survival after initial hepatectomy according to recurrence timing among patients with recurrence (n = 109).

Early recurrence was defined as recurrence within 12 months after hepatectomy, and late recurrence as recurrence occurring at or beyond 12 months.

## Supplementary Figure S2

Post-progression survival by recurrence site among patients with early recurrence

Organ-specific recurrence patterns were examined as exploratory supplementary analyses. Kaplan–Meier curves of post-progression survival (PPS) according to recurrence site among patients with early recurrence (<12 months) after initial hepatectomy for CRLM (liver only, n = 27; lung only, n = 15; liver + lung, n = 14; other extrahepatic, n = 22). PPS was defined as the interval from the date of the first recurrence after initial hepatectomy to death from any cause or the last follow-up. Numbers at risk are shown below the plot.

## Supplementary Figure S3

Three-month landmark analysis of post-progression survival according to salvage status in early recurrence

Legend

Three-month landmark analysis of PPS by salvageability among patients with early recurrence (<12 months) after initial hepatectomy for CRLM. Patients alive and under follow-up at 3 months after first recurrence were included, and time zero was set at the landmark. Salvageable by 3 months was defined as completion of salvage metastasectomy within 3 months after recurrence. The salvage date was defined as the earliest recorded salvage resection date after recurrence. Others were classified as non-salvageable by 3 months. PPS was estimated by the Kaplan–Meier method and compared using the log-rank test. Numbers at risk are shown below the plot.

## Supplementary Figure S4

*RAS* mutation rate according to recurrence timing and first recurrence site

a	b

Legend

(a) *RAS* mutation rate by recurrence timing (early recurrence <12 months vs late recurrence ≥12 months).

(b) *RAS* mutation rate by first recurrence site, stratified by recurrence timing.

Footnote
Bars indicate the proportion of *RAS*-mutant tumors. Denominators (n) indicate the number of patients with available *RAS* status in each subgroup. *RAS* status was unavailable in 11 patients with early recurrence; therefore, the organ-specific analysis among early recurrences was restricted to the 67 patients with available *RAS* data.
